# The major satellite DNA families of the diploid *Chenopodium album* aggregate species: Arguments for and against the “library hypothesis”

**DOI:** 10.1371/journal.pone.0241206

**Published:** 2020-10-27

**Authors:** Alexander Belyayev, Michaela Jandová, Jiřina Josefiová, Ruslan Kalendar, Václav Mahelka, Bohumil Mandák, Karol Krak

**Affiliations:** 1 The Czech Academy of Sciences, Institute of Botany, Průhonice, Czech Republic; 2 Department of Agricultural Sciences, University of Helsinki, Helsinki, Finland; 3 National Laboratory Astana, Nazarbayev University, Nur-Sultan, Kazakhstan; 4 Faculty of Environmental Sciences, Czech University of Life Sciences Prague, Praha, Suchdol, Czech Republic; University of Bari, ITALY

## Abstract

Satellite DNA (satDNA) is one of the major fractions of the eukaryotic nuclear genome. Highly variable satDNA is involved in various genome functions, and a clear link between satellites and phenotypes exists in a wide range of organisms. However, little is known about the origin and temporal dynamics of satDNA. The “library hypothesis” indicates that the rapid evolutionary changes experienced by satDNAs are mostly quantitative. Although this hypothesis has received some confirmation, a number of its aspects are still controversial. A recently developed next-generation sequencing (NGS) method allows the determination of the satDNA landscape and could shed light on unresolved issues. Here, we explore low-coverage NGS data to infer satDNA evolution in the phylogenetic context of the diploid species of the *Chenopodium album* aggregate. The application of the Illumina read assembly algorithm in combination with Oxford Nanopore sequencing and fluorescent *in situ* hybridization allowed the estimation of eight satDNA families within the studied group, six of which were newly described. The obtained set of satDNA families of different origins can be divided into several categories, namely group-specific, lineage-specific and species-specific. In the process of evolution, satDNA families can be transmitted vertically and can be eliminated over time. Moreover, transposable element-derived satDNA families may appear repeatedly in the satellitome, creating an illusion of family conservation. Thus, the obtained data refute the “library hypothesis”, rather than confirming it, and in our opinion, it is more appropriate to speak about “the library of the mechanisms of origin”.

## Introduction

Satellite DNA (satDNA) was discovered at the junction of the 1950s and 1960s via ultracentrifugation in a density gradient [1] and attracted increasing attention from researchers in recent years. Highly variable satDNA [2] that consists of long, late-replicating, non-coding arrays of tandemly arranged monomers is involved in various functions, ranging from chromosome organization and pairing to cell metabolism and modulation of gene functions [3–7]. Therefore, to better understand eukaryotic genome evolution and functioning, it is important to determine how and why satDNA varies among individuals and species, as there are clear links between satellites and phenotypes in a wide range of organisms [8].

The rapid quantitative and qualitative evolution of satellitome elements leads to the formation of complicated, difficult to decrypt species-specific profiles, and the best way to define satDNA formation patterns is to project satellitome dynamics on the evolution of a known biological system. Thus, in 1976 Salser et al. [9] suggested the “library hypothesis” based on rodent taxa, proposing that: “To explain the conservation of satellite sequences over long evolutionary periods during which they seem to appear and disappear many times, we have suggested a new model in which it is proposed that the rodents (and perhaps other mammalia) share a common library of satellite sequences. In each species, certain members of this library may be amplified and appear as major satellite peaks, while other satellite sequences are present at low levels undetectable in the analytical ultracentrifuge. According to this model, the rapid evolutionary changes undergone by satellite DNAs would be for the most part quantitative. Appearances of new satellites would usually represent amplification of one of the satellites already present at low level in the “library” rather than appearance de novo as in the original Southern (1970) model” [10]. According to this model, the rapid evolutionary changes experienced by satDNAs would be mostly quantitative. Although the library hypothesis has been confirmed by data from other living systems [11–14], this hypothesis does not address several important questions, such as (i) how novel satellites emerge and (ii) how libraries form and survive speciation-related repeatome purification [15] and subsequent concerted evolution, in addition to ignoring the phenomenon of the recurrent appearance of satellitome components from, for example, transposable elements (TEs) [7, 16].

To shed light on at least some of these problems, a recently developed method of next-generation sequencing (NGS) data assembly is useful. In this method, short DNA fragments are aligned and merged to reconstruct the original sequence [17], thus allowing the determination of the satDNA landscape. Here, we intended to analyze NGS Illumina data to reconstruct satellite evolution in the well-developed phylogenetic context of the Eurasian representatives of the *Chenopodium album* aggregate. The evolutionary history of this species complex has been shaped by extensive hybridization and polyploidization and was described in detail recently [18]. Eight basic evolutionary lineages represented by five extant and three extinct/unknown diploid taxa have been identified within this group. The diversification of the group started at the Miocene; however, most of the lineages formed later, close to the beginning of the Quaternary period [18]. Superimposed on this sequence of evolutionary events, parameters of the satellitome will reflect the proliferation of the complex over time and, consequently, the lineage-specific trends and patterns in the satDNA family’s evolution and amplification.

Given the long evolutionary history and wide distribution of *C*. *album* aggregate in various climatic regions, we hypothesized that differential satellitome dynamics may occur in diverse lineages. The “library hypothesis” would be supported by cases of long-term conservation of satellitome elements during evolution, while cases of elimination and/or the emergence of new elements would oppose it. Thus, we aimed to evaluate the satDNA landscape of the distinct evolutionary lineages recognized within the *C*. *album* aggregate that are represented by extant related diploid species. However, considered the limitations regarding the assembly of satellite repeats based on short read sequence data [16, 19]. Thus, an alternative assembly-free approach involving the use of ultralong Oxford Nanopore reads (ONs) was used to (i) confirm the results based on Illumina data and (ii) determine the length of different satellite repeat arrays and their association in the genome. The combination of these two types of data may indicate the vectors of species divergence at the molecular level with higher accuracy.

## Materials and methods

### Statement of permit requirements

No permits were required for this study.

#### Plant material, DNA extraction, library preparation, and Illumina sequencing

For both the preparation of the DNA libraries and cytogenetic experiments, plants of the following diploid species were used: *C*. *acuminatum* Willd., *C*. *bryoniifolium* Bunge, *C*. *ficifolium* Sm., *C*. *iljinii* Golosk., *C*. *pamiricum* Iljin, *C*. *suecicum* J. Murr, and *C*. *vulvaria* L., which represent the main *Chenopodium album* aggregate lineages (Table 1). For our research, we sampled genotypes that present average parameters for the lineage according to our previous work [18]. All plants were cultivated in the experimental garden of the Institute of Botany, Czech Academy of Sciences, Průhonice, Czech Republic (49.9917°N, 14.5667°E, ca. 320 m above sea level). Leaves were collected, and DNA was extracted using the DNeasy Plant Mini Kit (Qiagen, Hilden, Germany) according to the manufacturer’s instructions. For *in situ* hybridization experiments, the root tips of young roots were collected and fixed as described by Mandák et al. [20], then stored until use.

**Table 1:** The accessions and geographic origins of diploid *Chenopodium* species (2n = 2x = 18).

Species (accession number)	Genome	Locality	Genome Size Mbp
*C*. *acuminatum* (429–3)	D	China, Xinjiang, Altaj, Burqin	960
*C*. *bryoniifolium* (742–4)	A	Russian Federation, Primorski Krai, Nakhodka City District	2608
*C*. *ficifolium* (330–2)	B	Czech Republic, Slatina	1785
*C*. *iljinii* (433–9)	E	China, Xinjiang, Altaj, Hoboksar	1144
*C*. *pamiricum* (830–3)	E	Tajikistan, Gorno-Badakhshan autonomous region, Murghob District	1154
*C*. *suecicum* (328–10)	B	Czech Republic, Švermov	1775
*C*. *vulvaria* (771–1)	H	Iran, Ardabil, Meshgin Shahr	924

One individual per species was used for library preparation and NGS. One microgram of extracted DNA was sheared into fragments of approximately 500–600 bp using a Bioruptor Pico sonication device (Diagenode, Liège, Belgium). NEBNext adaptors for Illumina were ligated to the resulting fragments using the NEBNext Ultra DNA Library Prep Kit for Illumina (New England BioLabs, Ipswich, MA, USA) following the manufacturer’s instructions. The QIAquick PCR Purification Kit (Qiagen, Hilden, Germany) was used to clean unbound adapters from the samples and to concentrate the samples to a total volume of 30 μl. Thereafter, the samples were loaded into a 1% agarose gel in low-EDTA/TAE buffer. Fragments with sizes ranging from 500 to 750 bp were excised and purified using the Zymoclean Gel DNA Recovery Kit (Zymo Research, Irvine, CA, USA) and eluted into 20 μl of ddH2O, after which their concentration was estimated with a Qubit fluorometer using the Qubit HS Assay kit (Thermo Scientific, Waltham, MA, USA). The individual libraries (corresponding to individual species) were enriched and indexed with unique barcodes using PCR with NEBNext Q5 HotStart HiFi PCR Master Mix and NEBNext Multiplex Oligos for Illumina (New England BioLabs) according to the manufacturer’s instructions. The enriched libraries were purified twice using AMPure magnetic beads (Beckmann Coulter, Pasadena, CA, USA), with a bead:library ratio of 0.7:1 in the first purification and 1:1 in the second purification. The libraries were checked on 1% agarose gels after each purification step and concentration was measured using the Qubit HS Assay kit (Thermo Scientific) after the final purification step. The libraries of all seven species were pooled and sequenced in an Illumina MiSeq system at Macrogen Inc. to obtain 300 bp paired-end reads (genome coverage 0.4–0.9x, which is sufficient for the identification of major satDNA families). The Illumina data have been deposited in the NCBI Sequence Read Archive as BioProject PRJNA634444.

#### Assembly and contig screening for tandem repeats

For the processing of the Illumina NGS data and the identification of the colocalization of TEs and CficCl-61-40 satDNA family arrays in the genomes of all investigated species, Geneious Prime software version 2019.2.1 (https://www.geneious.com) was used [21]. The advantage of this assembler is that it produces large contigs. *De novo* assembly was performed with medium-low sensitivity, which is the best option for large numbers (e.g., 100,000 or more) of Illumina sequencing reads. The 1,000 longest contigs, excluding those from mitochondria, chloroplasts and rDNA, were analyzed using the following two publicly available online tools: tandem repeat finder (TRF) (https://tandem.bu.edu/trf/trf.html) [22] and the YASS genomic similarity tool (http://bioinfo.lifl.fr/yass/yass.php) [23], which enables searches for potential tandem organization. Contigs containing long satDNA arrays (> 30 monomers for minisatellites and > 5 monomers for tandem repeats) were selected manually. In parallel, the most common microsatellites for each species were determined. The selected contigs were checked for similarities with BLAST. For newly discovered satDNA families that did not show any similarities in the database, a consensus monomer was determined (S1 Table). Conservative motifs of 9–12 bp were distinguished within the consensus monomer for further *in silico* genome scanning to quantitatively determine the approximate content (Table 2). The genomes of the remaining species were also scanned with the determined conserved motifs to identify the presence of the newly discovered satDNA families. Scanning was performed with the “search for motifs” command of the GP program, with a maximum of zero nucleotide mismatches. There could be a multitude of dispersed repeats in the genome [24], but they are of low copy numbers and can be considered genetic noise in most cases. Therefore, the existence of arrays for a specific satDNA was crucial for the conformation of the presence of that satDNA family in the genome of a single species. The percentage of microsatellites in the genome was approximately estimated by the number of hits of (TTA)_6_ motif per 50,000 longest contigs in each species.

**Table 2:** Primers and conserved motifs for satDNA families.

DNA of species	satDNA family	Conserved motif	Forward primer	Reverse primer
*C*. *ficifolium*	f1*	TTTCATTTGA	TCAAACAAAGCTTTTTGAATC	TTGTTTGAATGTGTTTGACTTT
*C*. *acuminatum*	f2	GCATGTAGA	GCATGTAGAAAATGGGAATGC	AATCAAGCAAATTCGGCAAA
*C*. *vulvaria*	f3	AGCCATATA	AGCCATATATGCTCGTTTTCAA	TCATTGAAATGAATGAACTAACAATTC
*C*. *suecicum*	f4	AATGGAATC	CAAACAAAGCAAATGGAATCAA	TTGCTTTGGGAATTCGTTTC
*C*. *pamiricum*	f5	AAGGGGCTC	ACATCATCGCCCATCTAAGG	TGGTACCCCTTCGGGTTAAT
*C*. *acuminatum*	f6	TATGTTCTAAA	CCGGTAAGAACCCCACCT	AGAACATAAACAACCAAAAA
*C*. *pamiricum*	f7	GGAGCGGGC	CTTTCTGACCCAGCAAGGAG	GCGCTCCATCTCTCTGCAC
*C*. *pamiricum*	f8	CCCGTCTGT	TTACACAGATGGTGAAATAAAAATTAC	TGTAATACACAGACGGGCAAA
*C*. *acuminatum*	MS	(TTA)_6_	Synthetic probe	

* The primers and conserved motif for Family 1 (f1) were described previously by Belyayev et al. [19].

#### SatDNA family characterization

Based on the consensus sequences of contigs that contain newly determined satDNA families, primers were designed using FastPCR (https://primerdigital.com/fastpcr.html) [25]. The primer sequences are provided in Table 2. PCR was performed in a 25 μL reaction containing TopBio Plain PP Master Mix (TopBio, Vestec, Czech Republic), each primer at 0.2 mM and 10 to 50 ng of genomic DNA. The cycling conditions were as follows: 4 min at 95°C, followed by 35 cycles of 95°C for 30 s, a sequence-specific annealing temperature for 30 s and 72°C for 2.5 min, with a final extension at 72°C for 10 min. The PCR results were verified in 1% agarose gels. The PCR products of clusters were excised from the gels, cloned and sequenced at GATC Biotech (Konstanz, Germany) according to standard protocols.

**Fig 1:**
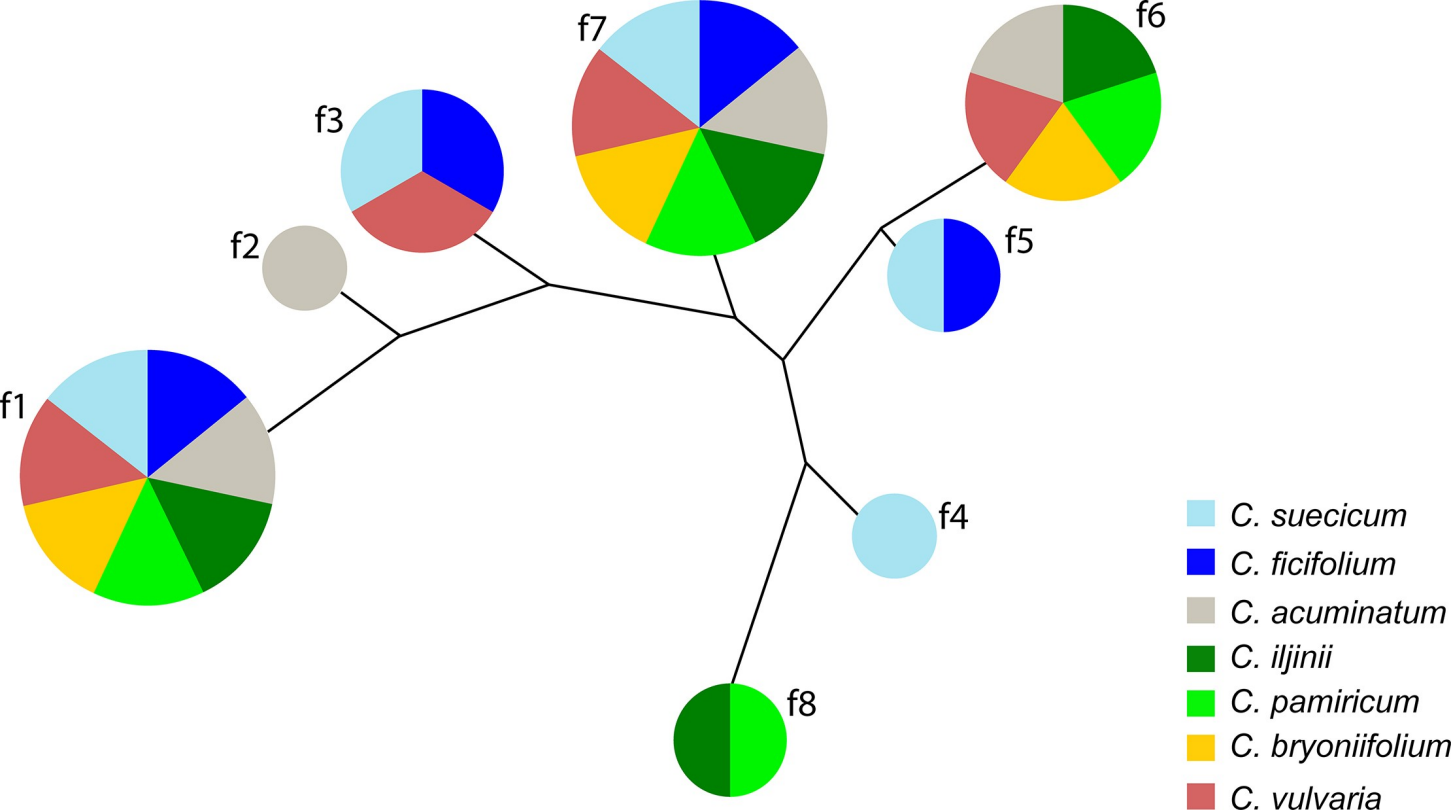
Relationships tree of the satDNA families identified in the genomes of diploid *C*. *album* aggregate species. The size of the circle is proportional to the number of species in whose genomes the satDNA family occurs.

For the reconstruction of the phylogenetic relationships among the analyzed monomers, multiple alignments were performed with ClustalW [26]. The phylogenetic relationships among the sequences were then reconstructed from the pairwise distance matrix [27]. The obtained distance matrix could be used to construct a phylogenetic tree via the minimum evolution method. The construction of the phylogenetic tree was performed with the MEGA program (Fig 1) [28].

#### Oxford nanopore sequencing

For Oxford nanopore sequencing, DNA of *C*. *acuminatum*, *C*. *pamiricum* and *C*. *suecicum* was used. DNA was fragmented by pipetting. The sequencing libraries were prepared from 1 μg of the partially fragmented DNA using Ligation Sequencing Kit SQK- LSK109 (Oxford Nanopore Technologies) following the manufacturer’s protocol. The DNA was treated with 2 μl of NEBNext FFPE DNA Repair Mix and 3 μl of NEBNext Ultra II End-prep enzyme mix in a 60 μl volume that also included 3.5 μl of both the FFPE and End-prep reaction buffers (New England Biolabs). The reaction was performed at 20°C for 5 min and 65°C for 5 min, followed by purification using a 1x volume of AMPure XP beads (Beckman Coulter). Subsequent steps, including adapter ligation using NEBNext Quick T4 DNA Ligase and library preparation for sequencing, were performed according to the provided protocols. Each library was loaded separately onto the FLO-MIN106 R9.4 flow cell and sequenced for 20 h. SatDNA family array searches were performed with the same algorithm employed for the assembled reads.

#### *In situ* probe preparation and FISH procedure

For the characterization of the chromosomal distribution of satDNA families, fluorescent *in situ* hybridization (FISH) experiments were performed. The root fixation, slide preparation, probe labeling and FISH procedures were performed as described by Mandák et al. [18]. The slides were examined and photographed with a Zeiss Axio Imager Z2 microscope system.

## Results

### The major satDNA families in the genomes of diploid *C*. *album* aggregate species

*In silico* TRF scanning results were applied for the identification of eight satDNA families and the most common microsatellites in the genomes of diploid *C*. *album* aggregate species (Tables 2 and 3). The consensus monomer sequences of the determined satDNA families are presented in S1 Table. In Fig 1, the phylogenetic relationships of the satDNA families determined in genomes of the diploid *C*. *album* aggregate species are presented. The characteristics of each of the identified satDNA families are also shown below.

**Table 3:** Occurrence of specific satDNA families in the genomes of diploid *C*. *album* aggregate species.

Spec. (genome)/satDNA fam.	f1	f2	f3	f4	f5	f6	f7	f8
*C*. *ficifolium* (B)	+	–	+	–	+	–	+	–
*C*. *suecicum* (B)	+	–	+	+	+	–	+	–
*C*. *iljinii* (E)	+	–	–	–	–	+	+	+
*C*. *pamiricum* (E)	+	–	–	–	–	+	+	+
*C*. *acuminatum* (D)	+	+	–	–	–	+	+	–
*C*. *bryoniifolium* (A)	+	–	–	–	–	+	+	–
*C*. *vulvaria* (H)	+	–	+	–	–	+	+	–

**Fig 2:**
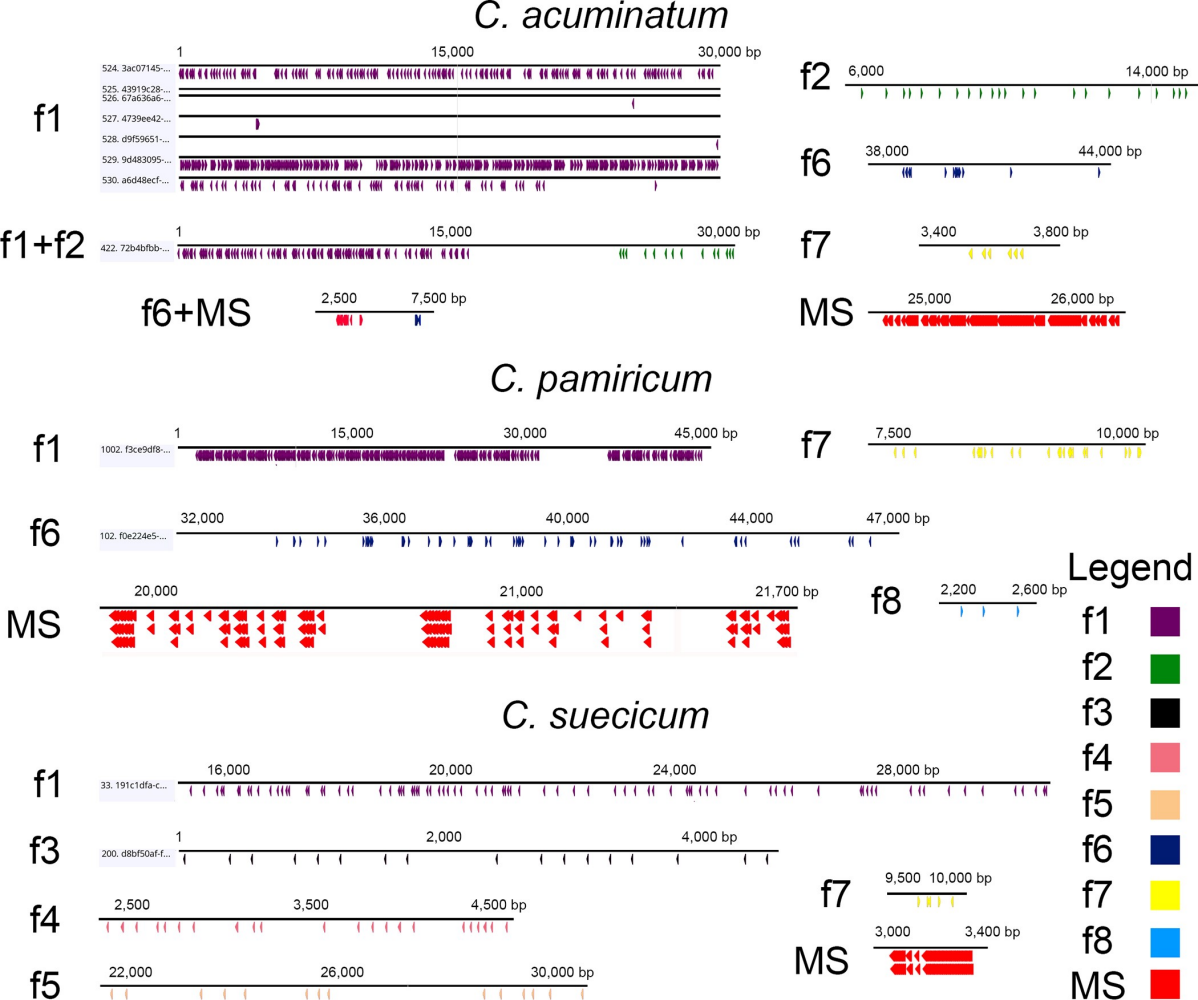
Arrays of satDNA families determined in genomes of three diploid *Chenopodium* species from ON reads. Screening was conducted on the basis of conserved motifs of satDNA family monomers (Table 2, S1 Table). satDNA family designation is the same as in the text, MS–microsatellites.

**Fig 3:**
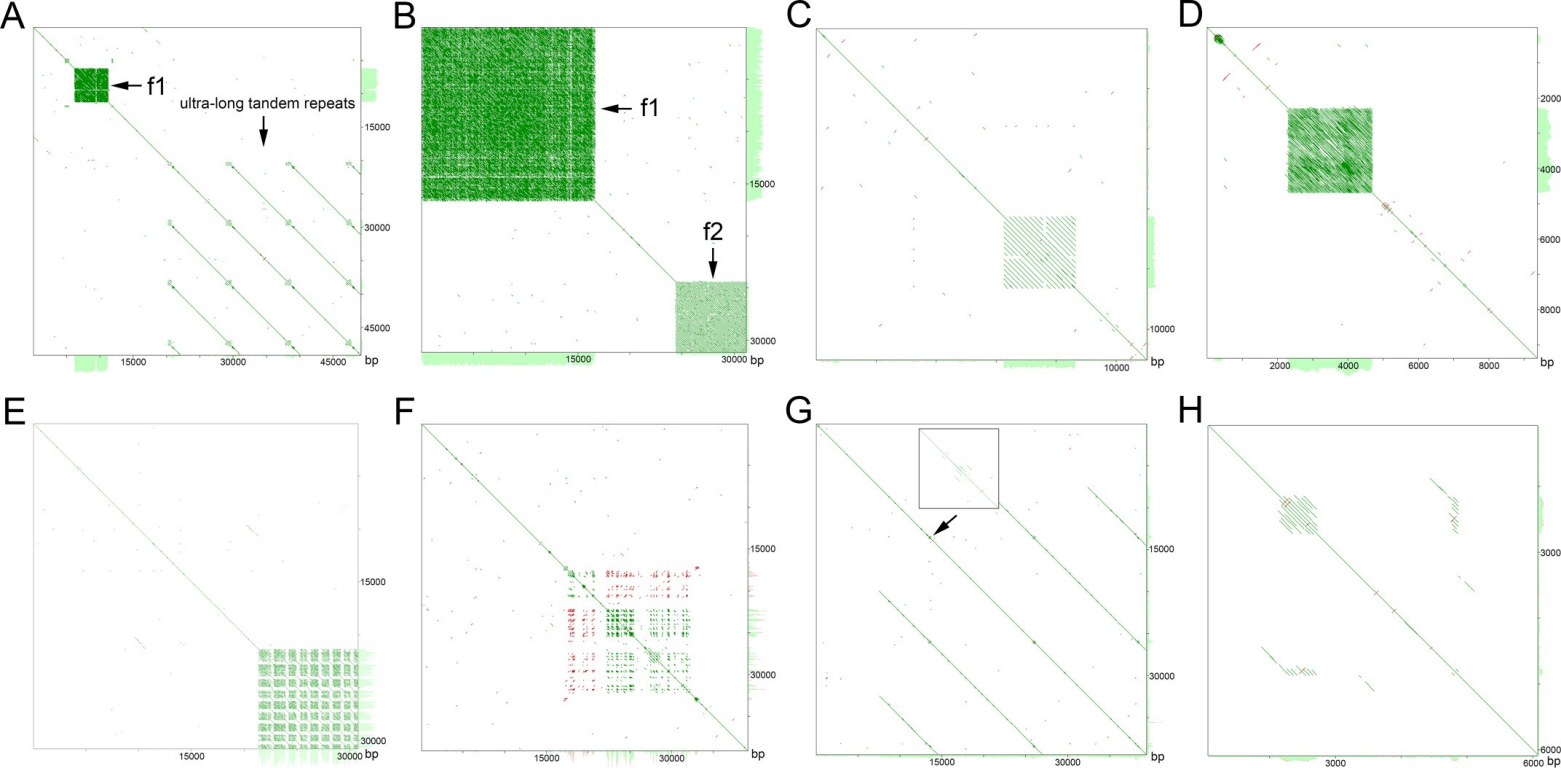
Self-to-self comparison of the ON reads displayed as dot plots (YASS program output), where parallel lines indicate tandem repeats (the distance between the diagonals is equal to the lengths of the motifs). (A) Array of the f1 satDNA family and long tandem repeats determined from read 17 of the ON reads for the *C*. *acuminatum* genome. (B) Colocalization of f1 and f2 satDNA family arrays in read 422 from the ON sequencing of the *C*. *acuminatum* genome. (C) Array of the f3 satDNA family in read 169 from the ON sequencing of the *C*. *suecicum* genome. (D) Array of the f4 satDNA family in read 210 from the ON sequencing of the *C*. *suecicum* genome. (E) Array of the f5 satDNA family in read 137 from the ON sequencing of the *C*. *suecicum* genome. (F) Array of the f6 satDNA family in read 2727 from the ON sequencing of the *C*. *pamiricum* genome. (G) Array of the f7 satDNA family in read 2621 from the ON sequencing of the *C*. *pamiricum* genome. (H) Array of the f8 satDNA family in read 5857 from the ON sequencing of the *C*. *pamiricum* genome.

**Fig 4:**
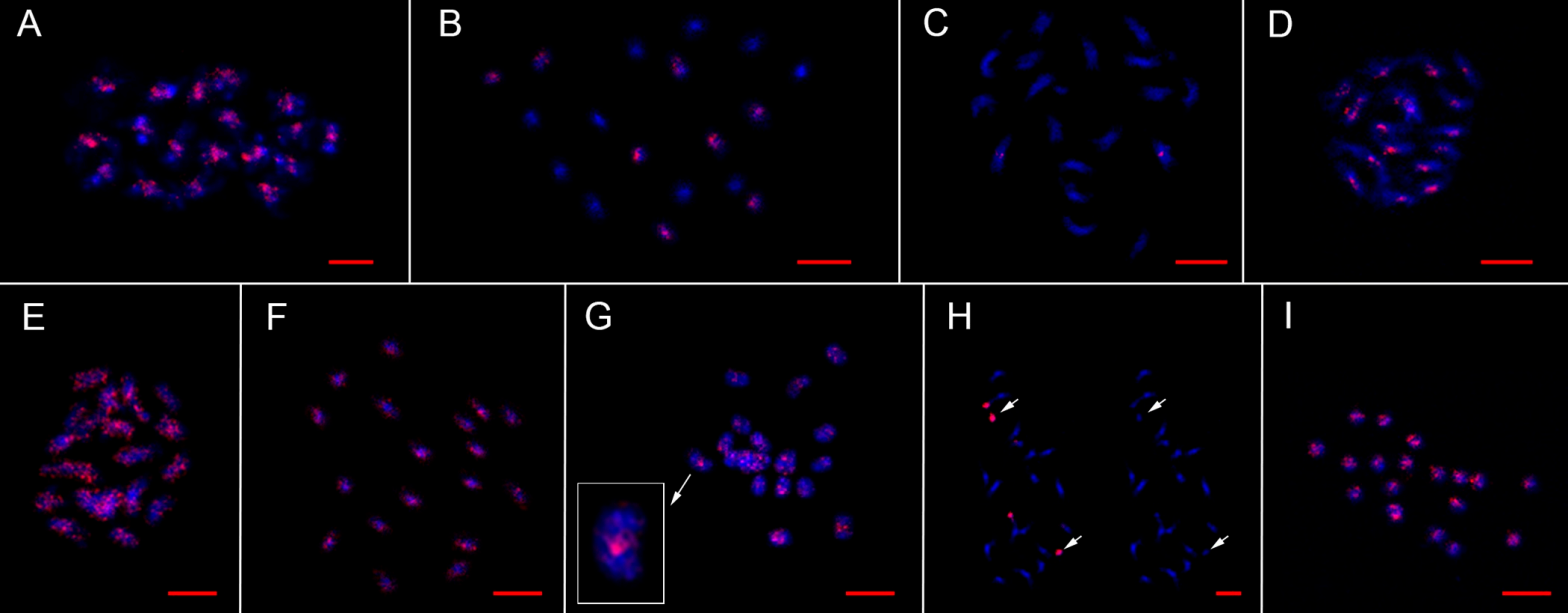
Chromosomal distribution of eight satDNA families. (A) FISH with the f1 probe on chromosomes of *C*. *acuminatum*. (B) FISH with the f2 probe on chromosomes of *C*. *acuminatum*. (C) FISH with the f3 probe on chromosomes of *C*. *suecicum*. (D) FISH with the f4 probe on chromosomes of *C*. *suecicum*. (E) FISH with the f5 probe on chromosomes of *C*. *suecicum*. (F) FISH with the f6 probe on chromosomes of *C*. *acuminatum*. (G) FISH with the f7 probe on chromosomes of *C*. *acuminatum*. An enlarged chromosome with a clear centromeric signal is shown in a separate box. (H) FISH with the f8 probe on chromosomes of *C*. *iljinii*. A metaphase plate with a red signal from the f8 satDNA family is shown on the left, and DAPI staining of the same metaphase plate is shown on the right. The smallest pair of chromosomes with major blocks is indicated by arrows. (I) FISH with an MS probe on chromosomes of *C*. *acuminatum*. All chromosomes were counterstained with DAPI. Bars represent 5 μm.

Family 1 (f1, same as the CficCl-61-40 satDNA family in Belyayev et al. [16, 19]) is the most common satDNA family that is present in the genomes of all studied species [19]. Its monomer length is approximately 40 bp (S1 Table). Each monomer contains a conserved TTTCATTTGA motif, which corresponds to the beginning of the parental CACTA-like transposable element fragment [16]. According to the ON data, f1 monomers form long arrays, reaching lengths of more than 40 kb, and have thousands of copies (Figs 2, 3A and 3B). According to the FISH data, f1 is distributed along all chromosomes, with an increased concentration in pericentromeric heterochromatin (Fig 4A).

Family 2 (f2) was identified in the genome of *C*. *acuminatum* (D-genome). Its monomer length is approximately 170 bp (S1 Table) and contains a conserved GCATGTAGA motif. f2 is associated with f1 (Fig 1) and is often located nearby. Read 422 from the ON sequencing (length 31 152 bp) of the *C*. *acuminatum* genome provides an example of f1 and f2 colocation (Fig 3B). FISH experiments revealed the distribution of f2 on four chromosome pairs of the *C*. *acuminatum* chromosome set (Fig 4B). The cloning of PCR-amplified DNA fragments showed 86.5–89.6% similarity with the assembled reference sequence. Sequences containing part of the f2 satDNA array were submitted to GenBank under accession number MT722943.

Family 3 (f3) was identified in B- and H-genome species, specifically in *C*. *ficifolium*, *C*. *suecicum* and *C*. *vulvaria*. f3 is similar to ChenSat-2d (GenBank: LR215738.1) detected in the genome of *C*. *quinoa* by Heitkam et al. [29], with monomer similarity of 92.35%. Its monomer length is approximately 170 bp (S1 Table) and contains a conserved AGCCATATA motif. The ON data showed the formation of arrays of up to 20 copies long (Figs 2 and 3C). FISH experiments revealed the distribution of f3 on one chromosome pair of the *C*. *suecicum* chromosome set (Fig 4C). Cloning of PCR-amplified DNA fragments from the genome of *C*. *vulvaria* showed 92.4% to 94.2% similarity with the assembled reference sequence. Sequences containing part of the f3 satDNA array were submitted to GenBank under accession number MT722944.

Family 4 (f4) is a species-specific satDNA family that is present in the genome of *C*. *suecicum*. Its monomer length is approximately 40 bp (S1 Table) and contains a conserved AATGGAATC motif. According to the ON data, f4 forms arrays of approximately 2500 bp in length (Figs 2 and 3D). FISH experiments revealed the distribution of f4 predominantly in pericentromeric heterochromatin of the *C*. *suecicum* chromosome set (Fig 4D). Cloning of PCR-amplified DNA fragments showed 90.0–95.0% similarity with the assembled reference sequence. The sequence containing part of the f4 satDNA array was submitted to GenBank under accession number MT722945.

Family 5 (f5) is a B-genome-specific satDNA family that is present in the *C*. *ficifolium* and *C*. *suecicum* genomes. It shows a partial match with clone ENA|LR215739|LR215739.1, with 95.83% similarity of corresponding fragments. Its monomer length is approximately 48 bp (S1 Table) and contains a conserved AAGGGGCTC motif. ON read 137 from the *C*. *suecicum* genome provides an example of an f5 array structure in which short arrays interspersed with short intervals form a combined major array of approximately 8000 bp in length (Figs 2 and 3E). FISH experiments revealed an evenly dispersed chromosomal distribution of f5 in the *C*. *suecicum* chromosome set (Fig 4E). Cloning of PCR-amplified DNA fragments showed 87.0–87.5% similarity with the assembled reference sequence. The sequence containing part of the f5 satDNA array was submitted to GenBank under accession number MT722946.

Family 6 (f6) is present in all explored diploids except for the B-genome species of *C*. *ficifolium* and *C*. *suecicum*. Its monomer length is approximately 21 bp (S1 Table) and contains the conserved TATGTTCTAAA motif. The YAAS output of ON read 2727 from the genome of *C*. *pamiricum* demonstrates a special structure of the f6 repeat arrays in which a conserved monomer of 21 bp is followed by highly variable fragments of different lengths (Fig 2). Thus, conserved repeats are distributed at different distances from each other, and the dot plot for this type of satDNA looks like a dotted square (Fig 3F). FISH experiments revealed an evenly dispersed chromosomal distribution of f6 in the *C*. *acuminatum* chromosome set (Fig 4F). Cloning of PCR-amplified DNA fragments from the *C*. *acuminatum* genome showed 80.0% - 95.5% similarity with the assembled reference sequence. The sequence containing part of the f6 satDNA array was submitted to GenBank under accession number MT722947.

Family 7 (f7) is the second widespread satDNA family that is present in the genomes of all studied species. Its monomer length is approximately 21 bp (S1 Table) and contains a conserved GGAGCGGGC motif. Positions from the first to thirteenth nucleotides are highly conserved in all explored species, but the rest of the monomer is variable even within a single genotype. ON read 2621 from the genome of *C*. *pamiricum* demonstrated that this satDNA family forms relatively short arrays (Figs 2 and 3G). Another finding from ON sequencing was that fragments of conserved retrotransposon domains (reverse transcriptase Cdd: cd01647) are very often in close proximity to f7 arrays. According to FISH data, f7 is distributed in centromeric regions and pericentromeric heterochromatin (Fig 4G). The cloning of PCR-amplified DNA fragments from the genome of *C*. *acuminatum* showed ~70.0% similarity with the assembled reference sequence. The sequence containing part of the f7 satDNA array was submitted to GenBank under accession number MT722948.

Family 8 (f8) is E-genome specific and is present in the genomes of *C*. *iljinii* and *C*. *pamiricum*. Its monomer length is approximately 60 bp (S1 Table) and contains a conserved CCCGTCTGT motif. ON reads 4586 and 5857 from the genome of *C*. *pamiricum* demonstrated that this satDNA family forms relatively short arrays (Figs 2 and 3H). FISH experiments revealed a clustered chromosomal distribution of f8 in the *C*. *iljinii* chromosome set (Fig 4H). There are four major clusters and two minor clusters on three pairs of chromosomes. The major clusters completely cover the two smallest chromosomes and are terminally located on one of the largest chromosome pairs. The minor clusters are located in the pericentromeric region of one chromosome pair. Cloning of PCR-amplified DNA fragments from the genome of *C*. *pamiricum* showed 85.0–93.3% similarity with the assembled reference sequence. The sequence containing part of the f8 satDNA array was submitted to GenBank under accession number MT722949.

Within the diverse microsatellites in the genomes of diploid *C*. *album* aggregate species, the TTA microsatellite is the most abundant. Species differ significantly in the *in silico*-determined density of TTA per genome unit. If TTA density in the genome of *C*. *acuminatum* is taken as 100%, the density in the genomes of the remaining species is as follows: *C*. *bryoniifolium—*144%, *C*. *ficifolium–* 141%, *C*. *iljinii–* 54%, *C*. *pamiricum–* 252%, *C*. *suecicum–* 156%, and *C*. *vulvaria–* 132%. ON sequencing shows that TTA microsatellites may form arrays of up to 1500 bp in length (Fig 2). According to FISH data, TTA microsatellites are distributed along all chromosomes of the investigated species, with an increased concentration in pericentromeric heterochromatin (Fig 4I).

One of the outcomes of ON sequencing was the discovery of ultralong tandem repeats in the genomes of the investigated species. Thus, together with the f1 tandem array, an ultralong tandem array with a monomer of approximately 13 kb is presented in Fig 3A. It should be emphasized that within this array, no TE-related conserved domains (even incomplete or nonfunctional domains) were found.

## Discussion

Application of the short read genome assembly algorithm in combination with ON sequencing allowed the estimation of the satDNA landscape of the genomes of the diploid *C*. *album* aggregate species. The obtained diverse set of satDNA families can be divided into several categories: group-specific, lineage-specific and species-specific. The group-specific families include two families, f1 and f7, both of which are connected with different TEs. The first is the highest copy-number satDNA family in the *Chenopodium* genome, which constitutes up to 3.8% of the total genomic DNA of the explored species [19]. According to our previous work [16], f1 appears to be a mixture of long-formed and diverged monomers of different lengths transmitted vertically during the process of evolution, plus novel uniform monomers of ~40 bp that were formed from the deletion derivatives of the parental CACTA-like element *Jozin*. At least a portion of the ancient monomers are far-diverged spin-offs and could be a source of the new, f1-related satDNA families (see below). In contrast, the newly formed f1 satDNA family monomers present a similar nucleotide composition in different species since they originated from the same fragment of the conserved CACTA-like element *tnp2* domain. This similarity may create an illusion of conservation; however, as noted previously, monomers arise repeatedly and independently in different lineages [16]. Perhaps this continuous replenishment of species genomes with new identical satDNA monomers could be a possible explanation for the phenomenon of the long-existing satellite families described in species such as mollusks [30].

Another group-specific satDNA family is f7, which is most likely also connected with TEs. Several arguments can be made in favor of this assumption. First, ON sequencing followed by the analysis of conserved domains showed the presence of retrotransposon fragments, particularly fragments or RT domains, in close proximity to f7 arrays. Second, a self-to-self comparison of the ON reads displayed as a dot plot revealed the presence of f7 short arrays at the ends of long tandem repeats that are most likely decayed TEs according to their lengths (Fig 3G). In this case, f7 satDNA monomers could be components of the LTRs of retrotransposons, although we did not find proper similarities with a specific TE. However, on the basis of FISH experiments with fragments of the f7 satDNA family as probes (Fig 4F), we propose that it could be a centromere-associated TE (a CRM retrotransposon, for example) as the FISH signals were strong (although the arrays were rather short) and were attributed predominantly to centromeric regions.

The lineage-specific satDNA families were f3, f5, f6 and f8. When these families were examined in a phylogenetic context, it became apparent that their origin does not fit a single model. For example, the f3 satDNA family was detected in the genomes of species that are separated by 11 Mya (clades B and H) [13]. Thus, it is logical to assume that (i) this tandem repeat is an ancient component that was present in the progenitors of all present-day lineages and that (ii) the plant genomes of clades A, D and E lost this repeat during the evolutionary process [11]. Heitkam et al. [29] reported a further decrease in the copy number of this satDNA family in the B-genome containing tetraploid *C*. *quinoa* due to polyploidization. The f3 family is related to the f1 family (Fig 1) and could be a HOR derivative of f1 that was formed long ago at the time of the initial genesis of the *C*. *album* aggregate. In contrast, f5, f6 and f8 satDNA families are much younger and arose during the process of the divergence of several clades, but the exact pathway of the formation of these families is questionable, especially considering the unusual structure of the f6 satDNA family arrays.

The species-specific satDNA families are f2 and f4 (note that lineage D is present only by a single diploid species of *C*. *acuminatum*). Species-specific repeats may present fluctuating copy numbers even within the species range and are often completely or almost completely eliminated during speciation [15]. Species-specific satDNAs most likely form as a result of a single event. Given the proximity of satDNA family f2 to f1 (Fig 1), it can be proposed with a high probability that the 170 bp f2 monomer is also an HOR derivative of a 40 bp minisatellite of f1 that was formed later than the monomer of the f3 satDNA family, no earlier than ~ 5 Mya [18], at a specific stage of *C*. *acuminatum* genome evolution, especially considering that this genome possesses a tendency for HOR unit formation [19].

Two other satDNA families that were discovered in the genomes of the investigated species were microsatellites and ultralong tandem repeats. The predominant microsatellite motif in the explored genomes was TTA. Although microsatellites are known to be highly variable in terms of copy number in the genomes of the analyzed diploid species of the *C*. *album* aggregate, the genomes could be divided into high-microsatellite-copy-number genomes, such as that of *C*. *pamiricum*, and low-copy-number genomes, such as that of as *C*. *iljinii*, with the other species presenting intermediate values. The large difference in the numbers of microsatellites in the genomes of closely related *C*. *pamiricum* and *C*. *iljinii* are of special interest because of the ecological separation of these species. These species are not in contact at present: Pamiricum-Himalayan-endemic *C*. *pamiricum* grows at 3000 m asl and higher but was probably much more widely distributed during the Glacial period, while *C*. *iljinii* is a steppe-semidesert species growing at lower altitudes with a wider distribution [18]. It is clear that different environments have driven the relatively rapid divergence of the genome composition (particularly microsatellite copy numbers) of these two species. Concerning the observed ultralong tandem repeats, they are most likely decayed TEs (Fig 3A). Conserved domains are no longer detectable in the array, but based on monomer lengths, it can be concluded that they are TE residues that have retained their tandem organization.

If all of the obtained data are considered from the “library hypothesis” perspective, these data more often refute rather than confirm this hypothesis. The “library hypothesis” indicates that related species share a common satDNA library; certain members of this library may be amplified and appear as major members in each species while other satellite sequences are present at low levels; and the rapid evolutionary changes experienced by satellite DNAs will be mostly quantitative [9]. However, the present-day satellitome of diploid *C*. *album* aggregate species contains eight major satDNA families that arose at different times and by different mechanisms. Thus, the group-specific satDNA families f1 and f7 are TE derivatives that may have originated repeatedly during the evolution of the group. Families f2 and f3 are most likely HOR units originating based on f1 monomers, but at different times and in different lineages. Moreover, the ancient family f3 is no longer found in the majority of diploid species, which indicates its complete elimination during the evolutionary process. Thus, once a satDNA element originates, it could be transmitted vertically and be stable over time, in agreement with the “library hypothesis” which presupposes the long-term existence of satellitome elements in the genome within a specific set of satDNAs, but it could also be eliminated, which contradicts the “library hypothesis”. The appearance of unique, novel species-specific satDNA families such as f2 and f4 in a certain stage of genome evolution also opposes the “library hypothesis”. Therefore, in our opinion, it is more appropriate to speak about “the library of the mechanisms of origin” than about “the common satDNA library”. The study of the genomes of polyploid species will provide more information on satDNA family stability over time and dynamics in the process of evolution.

## Supporting information

S1 Table Consensus monomers of eight satDNA families. (DOCX)

## References

[1] Kit S. Equilibrium sedimentation in density gradients of DNA preparations from animal tissues. Journal of molecular biology. 1961; 3, 711–716. 10.1016/s0022-2836(61)80075-2 14456492

[2] Wei KH-C, Lower SE, Caldas IV, Sless TJS, Barbash DA, Clark AG. Variable Rates of Simple Satellite Gains across the *Drosophila* Phylogeny. Molecular biology and evolution. 2018; 35(4), 925–941. 10.1093/molbev/msy005 29361128 PMC5888958

[3] Martienssen RA. Maintenance of heterochromatin by RNA interference of tandem repeats. Nature Genetics. 2003; 35(3), 213–214. 10.1038/ng1252 14593407

[4] Kloc A, Martienssen R. RNAi, heterochromatin and the cell cycle. Trends Genetics. 2008; 24(10), 511–517. 10.1016/j.tig.2008.08.002 18778867

[5] Mehrotra S, Goyal V. Repetitive sequences in plant nuclear DNA: types, distribution, evolution and function. Genomics, Proteomics & Bioinformatics. 2014; 12(4): 164–171. 10.1016/j.gpb.2014.07.003 25132181 PMC4411372

[6] Garrido-Ramos MA. SatDNA in plants: more than just rubbish. Cytogenetics and Genome Research. 2015; 146(2): 153–170. 10.1159/000437008 26202574

[7] Meštrović N, Mravinac B, Pavlek M, Vojvoda-Zeljko T, Šatović E, Plohl, M. Structural and functional liaisons between transposable elements and satellite DNAs. Chromosome Research. 2015; 23(3): 583–596. 10.1007/s10577-015-9483-7 26293606

[8] Lower SS, McGurk MP, Clark AG, Barbash DA. Satellite DNA evolution: old ideas, new approaches. Current opinion in genetics & development. 2018; 49, 70–78. 10.1016/j.gde.2018.03.003 29579574 PMC5975084

[9] Salser W, Bowen S, Browne D, El Adli F, Fedoroff N, Fry K, et al Investigation of the organization of mammalian chromosomes at the DNA sequence level. Federal Proceedings. 1976; 35, 23–35. 1107072

[10] Southern EM. Base sequence and evolution of guinea pig satellite DNA. Nature. 1970; 227, 794–798. 10.1038/227794a0 4317352

[11] Mestrović N, Plohl M, Mravinac B, Ugarković D. Evolution of satellite DNAs from the genus Palorus—experimental evidence for the ‘library’ hypothesis. Molecular biology and evolution. 1998; 15(8), 1062–1068. 10.1093/oxfordjournals.molbev.a026005 9718733

[12] Plohl M, Meštrović N, Mravinac B. Satellite DNA evolution. Genome dynamics. 2012; 7: 126–152. 10.1159/000337122 22759817

[13] Ruiz-Ruano FJ, López-León MD, Cabrero J, Camacho JPM. High-throughput analysis of the satellitome illuminates satellite DNA evolution. Scientific reports. 2016; 6: 28333 10.1038/srep28333 27385065 PMC4935994

[14] Palacios-Gimenez OM, Milani D, Song H, Marti DA, López-León MD, Ruiz-Ruano FJ, et al Eight Million Years of Satellite DNA Evolution in Grasshoppers of the Genus Schistocerca Illuminate the Ins and Outs of the Library Hypothesis. Genome biology and evolution. 2020; 12(3): 88–102. 10.1093/gbe/evaa018 32211863 PMC7093836

[15] Belyayev A. Bursts of transposable elements as an evolutionary driving force. Journal of Evolutionary Biology. 2014; 27(12): 2573–2584. 10.1111/jeb.12513 25290698

[16] Belyayev A, Josefiová J, Jandová M, Mahelka V, Krak K, Mandák B. (2020) Transposons and satellite DNA: on the origin of the major satellite DNA family in the *Chenopodium* genome. Mobile DNA, 11, 20 10.1186/s13100-020-00219-7 32607133 PMC7320549

[17] Von Bubnoff A. Next-Generation Sequencing: The Race Is On. Cell. 2008; 132(7): 721–723. 10.1016/j.cell.2008.02.028 18329356

[18] Mandák B, Krak K, Vít P, Lomonosova MN, Belyayev A, Habibi F, et al Hybridization and polyploidization within the *Chenopodium album* aggregate analyzed by means of cytological and molecular markers. Molecular Phylogenetics and Evolution. 2018; 129: 189–201. 10.1016/j.ympev.2018.08.016 30172008

[19] Belyayev A, Josefiová J, Jandová M, Kalendar R, Krak K, Mandák B. Natural History of a Satellite DNA Family: From the Ancestral Genome Component to Species-Specific Sequences, Concerted and Non-Concerted Evolution. International journal of molecular sciences. 2019; 20(5), 1201 10.3390/ijms20051201 30857296 PMC6429384

[20] Mandák B., Krak K., Vít P., Pavlíková Z., Lomonosova M. N., Habibi F., et al How genome size variation is linked with evolution within *Chenopodium* sensu lato. Perspectives in Plant Ecology, Evolution and Systematics. 2016, 23, 18–32. 10.1016/j.ppees.2016.09.004

[21] Kearse M, Moir R, Wilson A, Stones-Havas S, Cheung M, Sturrock S, et al Geneious Basic: an integrated and extendable desktop software platform for the organization and analysis of sequence data. Bioinformatics. 2012; 28(12), 1647–1649. 10.1093/bioinformatics/bts199 22543367 PMC3371832

[22] Benson G. Tandem repeats finder: a program to analyze DNA sequences. Nucleic Acids Research. 1999; 27(2), 573–580. 10.1093/nar/27.2.573 9862982 PMC148217

[23] Noe L, Kucherov G. YASS: enhancing the sensitivity of DNA similarity search. Nucleic Acids Research. 2005; 33, W540–W543. 10.1093/nar/gki478 15980530 PMC1160238

[24] Sultana N, Menzel G, Heitkam T, Kojima KK, Bao W, Serçe S. Bioinformatic and Molecular Analysis of Satellite Repeat Diversity in Vaccinium Genomes. Genes. 2020; 11(5) 10.3390/genes11050527 32397417 PMC7290377

[25] Kalendar R, Khassenov B, Ramankulov Y, Samuilova O, Ivanov KI. FastPCR: an in silico tool for fast primer and probe design and advanced sequence analysis. Genomics. 2017; 109(4–5): 312–319. 10.1016/j.ygeno.2017.05.005 28502701

[26] Vinga S, Almeida J. Alignment-free sequence comparison—a review. Bioinformatics. 2003; 19: 513–523. 10.1093/bioinformatics/btg005 12611807

[27] Edgar RC. MUSCLE: a multiple sequence alignment method with reduced time and space complexity. BMC Bioinformatics. 2003; 5, 113 10.1186/1471-2105-5-113 15318951 PMC517706

[28] Kumar S, Stecher G, Li M, Knyaz C, Tamura K. MEGA X: Molecular Evolutionary Genetics Analysis across Computing Platforms. Molecular Biology and Evolution. 2018; 35(6), 1547–1549. 10.1093/molbev/msy096 29722887 PMC5967553

[29] Heitkam T, Weber B, Walter I, Ost C, Schmidt T. Satellite DNA landscapes after allotetraploidisation of quinoa (*Chenopodium quinoa*) reveal unique A and B subgenomes. Plant Journal. 2020 10.1111/tpj.14705 31981259

[30] Plohl M, Petrović V, Luchetti A, Ricci A, Satović E, Passamonti M, et al Long-term conservation vs high sequence divergence: the case of an extraordinarily old satellite DNA in bivalve mollusks. Heredity. 2010; 104(6), 543–551. 10.1038/hdy.2009.141 19844270

