# Supplementary material for: The major satellite DNA families of the diploid Chenopodium album aggregate species: Arguments for and against the “library hypothesis”
Source: PLoS One. 2020 Oct 27;15(10):e0241206. doi: 10.1371/journal.pone.0241206 (PMC7591062; doi:10.1371/journal.pone.0241206)
Supplement: S1 Table — (DOCX) [file pone.0241206.s001.docx]

**S1 Table.** Consensus monomers of eight satDNA families detected in genomes of diploid *Chenopodium* species

| species | Consensus sequence |
| --- | --- |
| Family 1 (~40 bp) | |
| C. acuminatum | TTTCATTTGATTCAAAAAGCTTTGTTGAATGTATTTGAC |
| C. bryoniifolium | TTTCATTTGATTCAATAAGCTTTGTTGAATGCATTTGAC |
| C. ficifolium | TTTCATTTGATTCAAAAAGCTTTGTTTGAATGTGTTTGAC |
| C. iljinii | TTTCATTTGATTCAAAAAGCTTTGTTTGAATGTGTTTGAC |
| C. pamiricum | TTTCATTTGATTCAAAAAGCTTTGTTTGAATGTGTTTGAC |
| C. suecicum | TTTCATTTGATTCAAAAAGCTTTGTTTGAATGTGTTTGAC |
| C. vulvaria | TTTCATTTGATTCAATTAGCTTTGTTGAATGCATTTGAC |
| Family 2 (~170 bp) | |
| C. acuminatum | GCATGTAGAAAATGGGAACGCTTCTTCTCATTTTGCCCGAATTTGCTTGATTTTGGTTCTACAAGACCTTAAAACCTTATAACTTGCTTTGTTTTTGTGATTTCTTTGCTTAGGAAGTTATAAATACATATATTTGACATGATTCAAGCCTTTATAAGTTCATTAGAAAGA |
| C. bryoniifolium | NOT DETECTED |
| C. ficifolium | NOT DETECTED |
| C. iljinii | NOT DETECTED |
| C. pamiricum | NOT DETECTED |
| C. sosNot detectedwskyi | NOT DETECTED |
| C. suecicum | NOT DETECTED |
| C. vulvaria | NOT DETECTED |
| Family 3 (~170 bp) | |
| C. acuminatum | NOT DETECTED |
| C. bryoniifolium | NOT DETECTED |
| C. ficifolium | AGCCATATAGGCCAAATTTGGATGATTTTGGATGAGTGAGGGTTGAAAATTGACATATGTGCTTATTTCTCTTAATTTCTATCCTTAGGCACTTAAAAGAATTGATATTGGAATGTTAGAAGAAAGAACGAGTCCATTTGCTTGAAAACTTAGAAAATAGGCATGGTTTT |
| C. iljinii | NOT DETECTED |
| C. pamiricum | NOT DETECTED |
| C. strictum | NOT DETECTED |
| C. suecicum | AGCCATATAGGCCAAATTTGGATGATTTTGGATGAGTTAGGGTCGATATTTGACATATGTGCTTATTTCTCTTAATTTCTATCCTTAGGCACTTAAAAGAAATTGATATTGGAATGTTAGAAGAAAGAACCTTACATTTGCTTGAAAACTTAGAAAATAGGCATGGTTTT |
| C. vulvaria | AGCCATATATGCTCGTTTTCAACTAGTTTGATGCATATTGAACCTAAAATGTAGGAAATGTGAGTTTTAAAACTCTTTTCAAGGTGTAGAAGCTTATATATATGCATATAAAGGATGATTGAATTGTTAGTTCATTCATTTCAATGATAATATGAAATATAGGCAAGTAAGT |
| Family 4 (~40 bp) | |
| C. acuminatum | NOT DETECTED |
| C. bryoniifolium | NOT DETECTED |
| C. ficifolium | NOT DETECTED |
| C. iljinii | NOT DETECTED |
| C. pamiricum | NOT DETECTED |
| C. suecicum | AATGGAATCAAATAGAAGTGAAACAGATTCAAACAAAGCA |
| C. vulvaria | NOT DETECTED |
| Family 5 (~48 bp) | |
| C. acuminatum | NOT DETECTED |
| C. bryoniifolium | NOT DETECTED |
| C. ficifolium | AAGGGGCTCATTAGCCCTAAGGGGCGTGAGACACATCATCGCCCATCC |
| C. iljinii | NOT DETECTED |
| C. pamiricum | NOT DETECTED |
| C. suecicum | AAGGGGCTCATTAGCCCTAAAGGGCGTCAGACACATCATCGCCCATCT |
| C. vulvaria | NOT DETECTED |
| Family 6 (~21 bp) | |
| C. acuminatum | TATGTTCTAAATTATTTTTTTC |
| C. bryoniifolium | TATGTTCTAAATTTTTTTTTCC |
| C. ficifolium | NOT DETECTED |
| C. iljinii | TATGTTCTAAATATTTAATCA |
| C. pamiricum | TATGTTCTAAATTATGTGCT |
| C. suecicum | TATGTTCTAAAGTATTTCTAT |
| C. vulvaria | TATGTTCTAAAGTTTATACTT |
| Family 7 (~21 bp) | |
| C. acuminatum | GGAGCGGGCGCTCCCATAGAGA |
| C. bryoniifolium | GGAGCGGGCGCTCCTCATTGA |
| C. ficifolium | GGAGCGGGCGCTCCTGGCTGTC |
| C. iljinii | GGAGCGGGCGCTCCTCATTGTA |
| C. pamiricum | GGAGCGGGCGCTCCTAGCTGT |
| C. suecicum | GGAGCGGGCGCTCCTGGCCGT |
| C. vulvaria | GGAGCGGGCGCTCTATGTGGT |
| Family 8 (~60 bp) | |
| C. acuminatum | NOT DETECTED |
| C. bryoniifolium | NOT DETECTED |
| C. ficifolium | NOT DETECTED |
| C. iljinii | CCCGTCTGTGTATTACACAGATGGGCAAAATATAAATTACCAAGTCATTGACTTGGTTAT |
| C. pamiricum | CCCGTCTGTGTATTACACAGATGGTGAAATTAAATTTACTAAGTTTTAAACTTAGTTTG |
| C. suecicum | NOT DETECTED |
| C. vulvaria | NOT DETECTED |
